# A formal validation of a deep learning-based automated workflow for the interpretation of the echocardiogram

**DOI:** 10.1038/s41467-022-34245-1

**Published:** 2022-11-09

**Authors:** Jasper Tromp, David Bauer, Brian L. Claggett, Matthew Frost, Mathias Bøtcher Iversen, Narayana Prasad, Mark C. Petrie, Martin G. Larson, Justin A. Ezekowitz, Scott D. Solomon

**Affiliations:** 1 Saw Swee Hock School of Public Health, National University of Singapore & National University Health System, Singapore, Singapore; 2 Duke-NUS Medical School, Singapore, Singapore; 3 Cardiovascular Division, Brigham and Women’s Hospital, Harvard Medical School, Boston, MA USA; 4 Us2.ai, Singapore, Singapore; 5 British Heart Foundation Cardiovascular Research Centre, University of Glasgow, Glasgow, UK; 6 Department of Biostatistics, School of Public Health, Boston University, Boston, MA USA; 7 Division of Cardiology and Mazankowski Alberta Heart Institute, University of Alberta, Edmonton, AB Canada; 8 Canadian Vigour Centre, University of Alberta, Edmonton, AB Canada

**Keywords:** Cardiology, Cardiac device therapy, Machine learning

## Abstract

This study compares a deep learning interpretation of 23 echocardiographic parameters—including cardiac volumes, ejection fraction, and Doppler measurements—with three repeated measurements by core lab sonographers. The primary outcome metric, the individual equivalence coefficient (IEC), compares the disagreement between deep learning and human readers relative to the disagreement among human readers. The pre-determined non-inferiority criterion is 0.25 for the upper bound of the 95% confidence interval. Among 602 anonymised echocardiographic studies from 600 people (421 with heart failure, 179 controls, 69% women), the point estimates of IEC are all <0 and the upper bound of the 95% confidence intervals below 0.25, indicating that the disagreement between the deep learning and human measures is lower than the disagreement among three core lab readers. These results highlight the potential of deep learning algorithms to improve efficiency and reduce the costs of echocardiography.

## Abstract (web summary)

Deep learning can automate the interpretation of medical imaging tests. Here, the authors formally assess the interchangeability of deep learning algorithms with expert human measurements for interpreting echocardiographic studies.

## Introduction

Deep learning algorithms, a subset of machine learning algorithms, can analyze medical images more efficiently, with improved consistency and fewer errors than humans^1–9^. Previous studies showed that deep learning models could accurately diagnose different types of skin cancers^2^, identify metastases in breast cancer patients^4^ and interpret arrhythmia on the electrocardiogram^5^. Compared to human interpretation of medical images, deep learning models can provide faster, more efficient, and more reproducible results.

Echocardiography is the most commonly used imaging modality for assessing cardiac structure and function due to its low cost, utility, and safety^10^. Efforts have been made to standardize the acquisition and interpretation of echocardiographic images^11–15^, which generally require dozens of measurements following the acquisition of images. However, these measurements are time-consuming and subject to high inter-and intrareader variability and human error, even amongst specialists^13,16^. Several studies have shown that deep learning algorithms can classify echocardiographic images according to their specific view (e.g., apical 4 chamber [A4C], or parasternal long axis [PLAX])^17–20^, quantify cardiac volumes and assess cardiac systolic function^18,19,21–24^. We previously demonstrated the development and external validation of an automated deep learning-based workflow for the classification and annotation of echocardiographic videos and images^25^.

However, few adequately powered studies have compared the interchangeability of deep learning algorithms against expert human measurements for interpreting the echocardiogram. In this study, we perform a formally powered validation of an automated deep learning workflow against ‘gold-standard’ Echocore lab measurements.

## Results

### Participant characteristics

Supplementary Table 1 shows the characteristics of participants. The mean age of 600 participants was 57 (±16) years, 186 (69%) were women, and 421 (70%) had HFrEF. The mean systolic blood pressure was 120 (±17) mm Hg. The mean LVEF was 42% (±14%), the mean E/e’ was 12 (±7). The three readers were physicians with extensive echocardiography experience and worked in an echo-core laboratory, with a range of 5–15 years of experience. The analysis time per study on an eight-core CPU ranged from 0.3 to 9.3 min with a median of 1 min (interquartile range, 0.8–1.3 min).

### Primary outcome

Table 1 shows the average yield for each parameter. The absolute yield of study variables in the dataset measurable by all three human readers ranged from a high of 585 studies (left atrial end-systolic volume [LAESV]) to a low of 217 studies (tricuspid regurgitation [TR] velocity). The yield of study variables measured by all three human readers and deep learning workflow ranged from a high of 547 (Inter Ventricular Septal diameter [IVSd] and Left Ventricular Posterior Wall diameter [LVPWd]) to a low of 149 (TR Vmax). The yield proportions ranged from a high of 0.97 (right atrial area [RA] area in apical four-chamber [A4C]) to a low of 0.69 (TR Vmax), with an average of 0.88 across all 23 study parameters. Supplementary table 2 demonstrates the consequences of relaxing the confidence filters for TR Vmax, s’ lateral, and s’ septal, by turning all of them off, moving all automated “low confidence” measurements into “high confidence. Among TR Vmax signals with 3 human reads, automated workflow “low confidence” explains only 18% of missing reads. Relaxing the confidence threshold resulted in better yield and reduced IEC.

**Table 1:** Yield results of the primary endpoint

Measurement	*n* (3 reader sets)	*n* (Automated)	Yield proportion	Yield (95% CI)	
				Lower	Upper
IVSd	584	547	0.94	0.92	0.95
LVIDd	579	550	0.95	0.93	0.96
LVIDs	572	537	0.94	0.92	0.95
LVPWd	579	547	0.94	0.93	0.96
LVEDV MOD biplane	583	534	0.92	0.89	0.93
LVESV MOD biplane	583	535	0.92	0.9	0.93
LVEF MOD biplane	583	531	0.91	0.89	0.93
LAESV MOD biplane	585	507	0.87	0.84	0.89
RA area A4C (s)	497	480	0.97	0.95	0.98
RVIDd	412	372	0.9	0.88	0.92
LVSV MOD biplane	583	533	0.91	0.89	0.93
MV-Adur	371	326	0.88	0.85	0.9
MV-E	560	508	0.91	0.88	0.92
MV-A	494	433	0.88	0.85	0.9
DecT	501	446	0.89	0.86	0.91
e’ lateral	547	458	0.84	0.81	0.86
e’ septal	541	490	0.91	0.88	0.92
E/e’ mean	511	388	0.76	0.73	0.79
a’ lateral	492	408	0.83	0.8	0.85
a’ septal	485	430	0.89	0.86	0.91
s’ lateral	547	445	0.81	0.78	0.84
s’ septal	549	459	0.84	0.81	0.86
TR Vmax	217	149	0.69	0.63	0.73

*DecT* deceleration time of early diastolic MV transmitral flow, *IVSd* interventricular septal diameter end diastolic, *LAESV MOD biplane* left atrial end systolic volume biplane calculation based on method of discs, *LVEDV MOD biplane* left ventricular end diastolic volume biplane calculation based on method of discs, *LVEF MOD biplane* left ventricular ejection fraction biplane based on method of discs, *LVESV MOD biplane* left ventricular end systolic volume biplane calculation based on method of discs, *LVIDd* left ventricular internal diameter at end diastole, *LVIDs* left ventricular internal diameter at end systole, *LVPWd* left ventricular posterior wall thickness measured end diastolic, *LVSV MOD biplane* left ventricular stroke volume biplane calculation based on method of discs, *MV-A* late diastolic transmitral flow, *MV-Adur* duration of late diastolic transmitral flow, *MV-E* early diastolic transmitral flow, *RA area a4c* right atrial area at end systole in A4C, *RVIDd* right ventricular end diastolic internal diameter, *TR Vmax* tricuspid regurgitation maximum velocity.

**Fig. 1:**
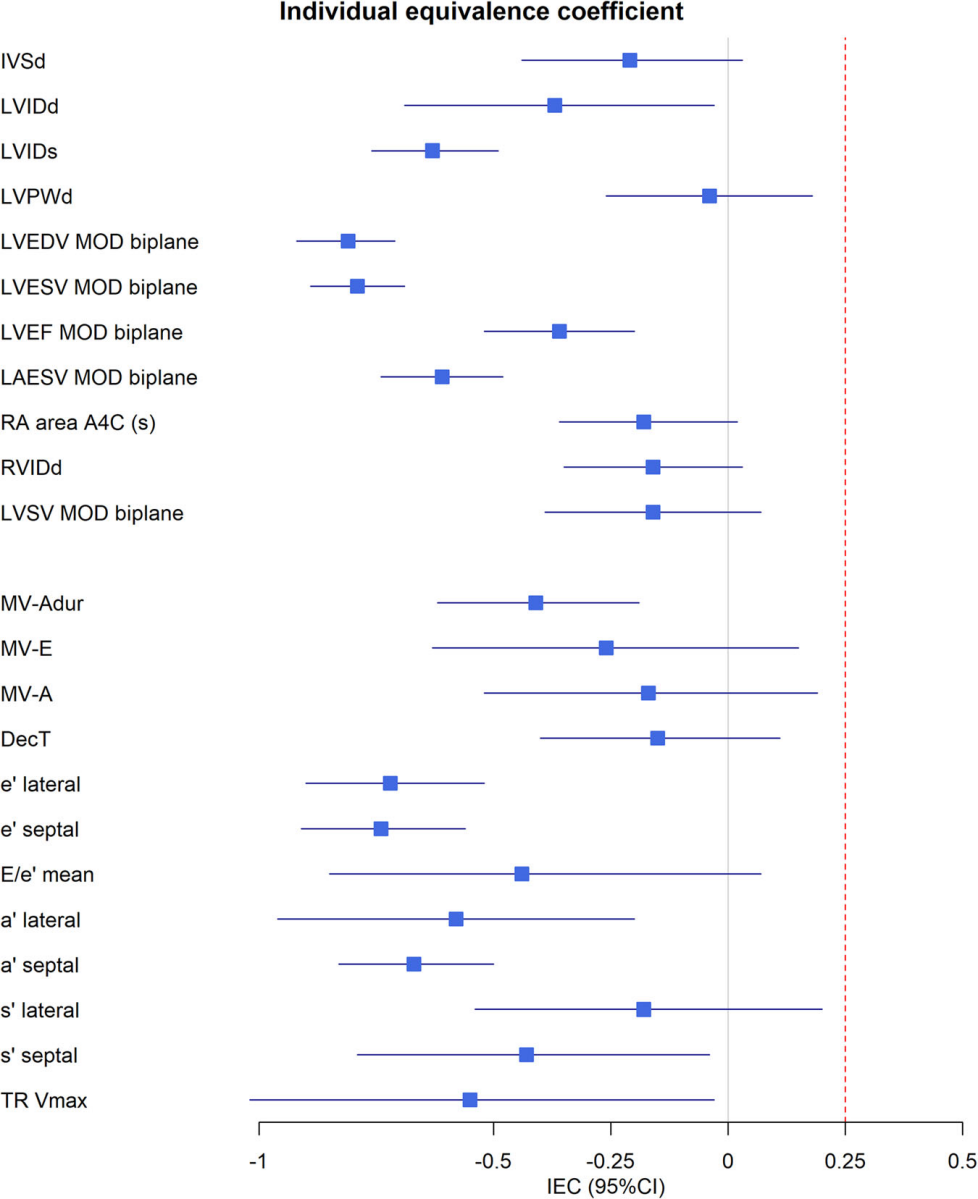
Forest plot showing the individual equivalence coefficients for twenty-three parameters. The blue box depicts the average individual equivalence coefficient. The error bars depict the upper and lower limits of the 95% confidence interval. The red line indicates the pre-determined non-inferiority criterion of 0.25 for the upper bound of the 95% confidence interval. Abbreviations and N (number of studies): a’ lateral (*N* = 408); a’ septal (*N* = 430); e’ lateral (*N* = 458); e’ septal (*N* = 490); E/e’ (*N* = 388); DecT, deceleration time of early diastolic MV transmitral flow (N = 446); IVSd, interventricular septal diameter end diastolic (*N* = 547); LAESV MOD biplane, left atrial end systolic volume biplane calculation based on method of discs (*N* = 507); LVEDV MOD biplane, left ventricular end diastolic volume biplane calculation based on method of discs (*N* = 534); LVEF MOD biplane, left ventricular ejection fraction biplane based on method of discs (*N* = 531); LVESV MOD biplane left ventricular end systolic volume biplane calculation based on method of discs (*N* = 535); LVIDd, left ventricular internal diameter at end diastole (*N* = 550); LVIDs left ventricular internal diameter at end systole (*N* = 537); LVPWd, left ventricular posterior wall thickness measured end diastolic (*N* = 547); LVSV MOD biplane, left ventricular stroke volume biplane calculation based on method of discs (*N* = 533); MV-A, late diastolic transmitral flow (*N* = 433); MV-Adur, duration of late diastolic transmitral flow (*N* = 326); MV-E, early diastolic transmitral flow (*N* = 508); RA area a4c, right atrial area at end systole in A4C (*N* = 480); RVIDd, right ventricular end diastolic internal diameter (*N* = 372); s’ lateral (*N* = 445); s’ septal (*N* = 459); TR Vmax, tricuspid regurgitation maximum velocity (N = 149).

**Fig. 2:**
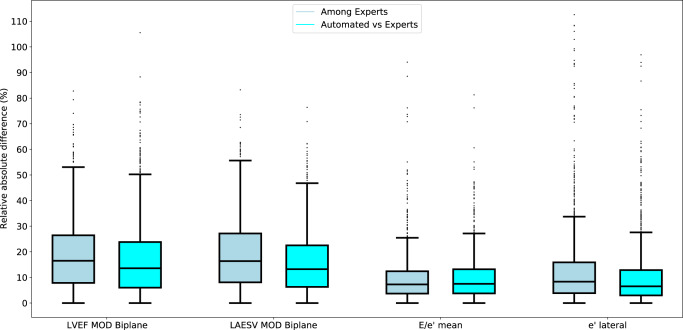
Box plot showing the relative absolute differences for four key parameters among expert sonographers and between automated readings and expert sonographers. Relative absolute difference among humans (dark blue) and between automated measurements and humans (light blue). The box plots centre line refers to the median. The box’ bounds reflect the 25^th^ and 75^th^ percentile. The distance between the box’ bounds and whiskers reflect the 25th or 75th percentile plus 1.5 times the interquartile range (i.e., the difference between the 25th and 75th percentile. Abbreviations and N (number of studies): E/e’ (*N* = 388); e’ lateral (*N* = 458); LAESV, left atrial end systolic volume (*N* = 507); LVEF, left ventricular ejection fraction (*N* = 531).

Figure 1 shows the results of the primary outcome. A mean IEC of −0.25, means that the variability (i.e., differences) between automated and human measurements were 25% lower than the variability among humans. A mean IEC of 0.25 means that the variability between automated and human measurements were 25% higher than the variability among humans. The mean IEC ranged from −0.04 for left ventricular posterior wall diameter to −0.81 for left ventricular diastolic volume. The upper 95% confidence interval fell below the prespecified success criterion of 0.25 for all 23 prespecified parameters, ranging from 0.20 (s’ lateral) to −0.71 (Left Ventricular Ejection Fraction [LVEF]). The relative absolute differences among humans and between automated and human measurements for key measurements are shown in Fig. 2. The relative absolute differences for LVEF, LAESV, E/e’ and e’ lateral were similar or lower for automated versus human experts than among human experts. Supplementary Table 3 shows that automated measurements were equivalent or superior to individual expert measurements.

### Secondary outcomes

Table 2 shows the exploratory outcomes. The ICC reflects the within-patient correlations between automated and human expert measurements or the correlations among human experts. The ICC for all comparisons (e.g., automated versus each human expert and all human experts versus each other) improved for all measurements when we added the comparisons between the automated and each human expert reader to comparisons among human expert readers. The ICCs automated versus human measurements were higher than the ICC among human experts (Supplementary Table 4).

**Table 2:** Results of secondary endpoints

Measurement	ICC		MAD		wCV		RMSE		R	LOA
	Automated + human readers	Human readers	Automated + human readers	Human readers	Automated + human readers	Human readers	Automated + human readers	Human readers	Automated versus human	Automated versus human
IVSd (mm)	0.63	0.61	1.15	1.2	11.33	11.55	1.12	1.15	0.66	0.33 ± 2.94
LVIDd (mm)	0.89	0.88	2.77	2.97	5.42	5.68	2.82	2.97	0.91	0.68 ± 7.29
LVIDs (mm)	0.91	0.89	2.95	3.6	7.44	8.11	3.13	3.41	0.93	−0.24 ± 7.80
LVPWd (mm)	0.62	0.63	1.16	1.16	11.11	11.22	1.09	1.09	0.61	−0.20 ± 2.98
LVEDV MOD biplane (ml)	0.83	0.79	21.17	27.6	17.93	20.34	22.39	25.09	0.88	−6.32 ± 52.08
LVESV MOD biplane (ml)	0.86	0.82	15.57	19.81	23.25	26.09	17.79	19.87	0.89	−1.47 ± 42.65
LVEF MOD biplane (%)	0.77	0.76	6.73	7.62	14.97	15.81	6.38	6.69	0.79	−1.23 ± 16.60
LAESV MOD biplane (ml)	0.85	0.82	9.24	11.44	16.91	18.43	9.89	10.74	0.88	−0.74 ± 24.75
RA area A4C (s) (cm^2^)	0.89	0.89	1.82	1.86	12.02	12.49	1.76	1.8	0.91	−0.95 ± 4.39
RVIDd (mm)	0.58	0.57	4.89	5.3	13.57	14.08	4.52	4.62	0.64	−2.11 ± 11.54
LVSV MOD biplane (ml)	0.48	0.4	12.49	13.21	23.69	24.82	11.5	11.74	0.58	−4.99 ± 29.60
MV-Adur (ms)	0.37	0.34	29.93	32.91	18.67	19.75	28.55	30.15	0.41	−1.34 ± 74.28
MV-E (cm/s)	0.96	0.96	4.46	4.62	6.55	6.81	5.05	5.22	0.97	−1.72 ± 13.07
MV-A (cm/s)	0.97	0.97	3.97	4.27	6.67	6.86	4.51	4.61	0.97	−1.75 ± 11.72
DecT (ms)	0.48	0.5	33.76	36.29	17.06	17.14	33.42	34.04	0.48	11.12 ± 88.10
e’ lateral (cm/s)	0.93	0.92	0.8	1.01	10.56	11.68	0.95	1.05	0.95	−0.05 ± 2.33
e’ septal (cm/s)	0.92	0.91	0.57	0.7	11.04	12.25	0.73	0.81	0.94	−0.02 ± 1.78
E/e’ mean	0.94	0.93	1.26	1.28	14.47	15.25	1.68	1.78	0.95	0.28 ± 4.31
a’ lateral (cm/s)	0.89	0.87	0.65	0.82	9.55	10.36	0.78	0.84	0.91	−0.09 ± 1.94
a’ septal (cm/s)	0.91	0.89	0.55	0.69	8.61	9.45	0.63	0.69	0.92	−0.06 ± 1.55
s’ lateral (cm/s)	0.95	0.95	0.51	0.54	7.23	7.45	0.53	0.55	0.96	−0.18 ± 1.39
s’ septal (cm/s)	0.94	0.93	0.38	0.43	7.03	7.46	0.41	0.43	0.94	−0.08 ± 1.06
TR Vmax (m/s)	0.91	0.89	0.13	0.15	6.03	6.53	0.16	0.17	0.93	−0.05 ± 0.39

*DecT* deceleration time of early diastolic MV transmitral flow, *IVSd* interventricular septal diameter end diastolic, *LAESV MOD biplane* left atrial end systolic volume biplane calculation based on method of discs, *LVEDV MOD biplane* left ventricular end diastolic volume biplane calculation based on method of discs, *LVEF MOD biplane* left ventricular ejection fraction biplane based on method of discs, *LVESV MOD biplane* left ventricular end systolic volume biplane calculation based on method of discs, *LVIDd* left ventricular internal diameter at end diastole, *LVIDs* left ventricular internal diameter at end systole, *LVPWd* left ventricular posterior wall thickness measured end diastolic, *LVSV MOD biplane* left ventricular stroke volume biplane calculation based on method of discs, *MV-A* late diastolic transmitral flow, *MV-Adur* duration of late diastolic transmitral flow, *MV-E* early diastolic transmitral flow, *RA area a4c* right atrial area at end systole in A4C, *RVIDd* right ventricular end diastolic internal diameter, *TR Vmax* tricuspid regurgitation maximum velocity.

The MAD reflects the absolute deviation between automated and human expert measurements. The average MAD for all comparisons decreased when we added the comparisons between the automated and each human expert reader to the comparisons among human expert readers (Table 2). The MAD between automated and individual human measurements was comparable or lower than the MAD among human experts (Supplementary Table 5).

The wCV reflects the within-patient variability of individual measurements relative to the within-patient mean. The wCV improved (decreased) for all measurements when automated measurements were included (Table 2). The RMSE reflects the spread of the residuals: the lower the RMSE, the better the agreement among the different measurements. The RMSE improved (decreased) when the total comparisons included automated versus human measurements on top of the comparisons among experts, relative to when all comparisons only included expert measurements (Table 2). Supplementary Tables 6 and 7 and show that the wCV and RMSE, respectively, were superior for automated versus human measurements relative to the wCV and RMSE between individual experts.

The correlation between automated and human expert measurements ranged from 0.41 for mitral valve (MV) A duration to 0.97 for MV-E and MV-A (Table 2). The correlations between automated and human expert measurements were higher than those among human readers (Supplementary Table 8).

The LOA between automated and human expert measurements ranged from 0.05 ± 0.39 for TR Vmax to 11.12 ± 88.10 for Deceleration time but showed generally good agreement between automated and human measurements. Supplementary Fig. 1 shows the Bland-Altman graphs for LVEF, LAESV, e’ lateral and E/e’ mean.

In secondary analyses, the ICC for monoplane measurements of all available images or videos passing the view confidence threshold ranged from 0.74 for LVEF to 0.96 for IVSd (Supplementary table 9)

## Discussion

This study demonstrated that the differences between deep learning-based measurements of and human experts of echocardiographic images are smaller or similar to differences in measurements among human expert interpretation of the same image. The mean absolute deviation between automated and human expert measurements was smaller than the difference among human experts for most parameters investigated. The median analysis time of a full echocardiographic study was only 1 min. Our results suggest that echocardiographic measurements performed by deep learning algorithms may be interchangeable with human expert assessment. These results emphasise the potential of deep learning algorithms to automate the tedious assessment of echocardiographic measurements, which can help increase access to-and reduce costs of echocardiography.

In the echo core lab, the sonographer or physician commonly selects the highest quality image/video to annotate based on his/her expertise. The automated workflow automated and standardized image/video selection by choosing only those images with the highest output probability of the view selection CNN. Our study compared deep learning based automated measurements with human sonographers on a study level. However, this was not a beat-for-beat comparison. There might be differences observed between expert sonographers and the automated workflow in a beat-for-beat comparison. Previous studies demonstrated the, albeit low, presence of intra-observer variability for most 2D volumes^26–28^ and Doppler measurements^26,29^. For example, in Frikha et al. the intra-observer ICCs was as low as 0.89 for LVEF^26^. The automated workflow selects the same video and performs the same measurement on re-analysis. However, this also means that the larger measurement variability among humans than between automated and human measurements could be explained by differences in video or beat selection by the human sonographers.

Our study showed that differences in measurements between human experts was often more significant than the differences between deep learning and human expert measurements. Previous studies using deep learning for echocardiography showed that deep learning algorithms could automatically interpret cardiac volumes^18^ and LVEF^18,19^. However, only a few studies tested the performance of their algorithms in external datasets. We previously showed that a deep learning algorithm successfully measured cardiac volumes, ejection fraction and Doppler measurements—with high correlation with human measurements—in external datasets, not used to train the algorithms^25^. The ICC was low for some of the linear (IVSd, LVPWd, RVIDd) and Doppler (MV A dur, DecT) measurements among humans. This could be explained by differences in selection of beats or videos by different sonographers. Our previous study showed that automated measurements from our deep learning-based workflow showed good agreement with locally measured values in a curated dataset from Canada, a real-world dataset from Taiwan and the US-based EchoNet-Dynamic dataset, which were analysed retrospectively^25^. Few fully powered deep learning studies exist in medical imaging^30^, which remains an important unmet need to demonstrate usability. In the present study, the point estimates of the IEC were below 0 for all parameters, this suggests that the disagreement among the three sonographers was larger than the disagreement between each individual sonographer and the automated workflow. However, reasons for disagreement between automated and human measurements could have been disagreement in the selection of videos, beats, frame or annotations among the trained sonographers and automated workflow, or poor performance of the CNNs. In our previous study, we found that, after blinded review of the top 15 outliers with the highest disagreement between clinical (manual) and automated measurements, sonographers preferred the automated over the manual measurement for most of the outliers^25^. In the present study, the yield was lower for some of the parameters, such as TRV max or s’ lateral and septal, which might have been caused by the quality control threshold. Indeed, when we relaxed the quality control threshold the yield increased but the IEC decreased for these parameters. These results emphasized the need for including some of these decision rules thus prioritizing reliability over yield as a decision support tool. Our study extends our previous work by formally testing the agreement of deep learning measurements of the echocardiogram with expert human measurements. Because the echocardiograms in the current study came from an echo core lab, the yield of the algorithms was higher in the present study than in our previous work.

Deep learning algorithms have shown the potential to substitute or supplement medical practitioners in repetitive tasks^2–4^. Deep learning algorithms can automatically detect lymph node metastases in women with breast cancer^4^, diabetic retinopathy on retinal fundus photographs^3^, or skin cancer^2^, with similar or superior accuracy compared to human experts. In echocardiography, previous attempts showed the potential of deep learning algorithms to automate the measurement of cardiac volumes and Doppler measurements^18,19,21–24^. A recent meta-analysis highlighted that only nine out of 81 studies validated deep learning algorithms against human experts. None of the studies was formally powered for the comparisons using an established protocol^30^. Therefore, our study is an important step forward and among the first studies powered to test the agreement of deep learning measurements against expert human measurements in a controlled setting.

Our study has several limitations. All included echocardiographic studies were of investigational grade quality. However, in a previous publication we showed good agreement between automated and ‘real-world’ clinical measurements. Nevertheless, the yield and performance of the automated workflow might be affected by the quality of the videos and images^25^. Our automated workflow includes decision rules to prevent reading low-quality images and providing low-quality results, as earlier published^25^. Therefore, the yield of our workflow might be lower in clinical practice. Second, our study did not include patients with heart failure with preserved ejection fraction (HFpEF) and atrial fibrillation. However, previous results showed that our algorithms perform equally well in patients with atrial fibrillation, HFrEF and HFpEF^25^. Therefore, it is unlikely that we would have observed a difference in our study.

The presented deep learning workflow can augment clinical care in several important ways. First, deep learning algorithms can augment the work of practicing cardiologists and sonographers. An intuitive user interface would allow the human reader to adjust the automated annotations directly on still images, reducing time spent on repetitive annotations while guaranteeing human control over output and quality. Second, deep learning algorithms can reduce the effects of intra-observational differences because frame and video selection are standardized. However, future challenges remain. The proposed automated workflow has not been validated for pediatric patients. Furthermore, our study only included patients in sinus rhythm. Our previous study suggested limited differences in the agreement between automated and manual measurements in patients with and without atrial fibrillation^25^. Together, our results show the potential of deep learning algorithms to democratize access to expert measurements and interpretation of the echocardiogram in settings with limited resources or expertise.

## Methods

### The deep learning workflow

Details on the design and development of the deep learning workflow were published previously^25^. Supplementary Fig. 2 shows an overview of the automated workflow. First, the DICOM tag is used to identify Doppler modalities from 2D videos. 2D videos and Doppler images are then parsed through two separate CNNs classifying them into their respective views (e.g., A2C, PLAX, A4C) and images (e.g., PW Doppler, CW Doppler, etc). An additional unsupervised clustering algorithm is used to classify 2D images. The probabilities of the CNN and clustering algorithm for 2D videos are then averaged. Furthermore, for each 2D video, the probability is averaged over the frames. Videos and images which do not reach a view-dependent probability threshold are considered of “poor view quality” and excluded from analysis. The remaining videos and Doppler images are then classified based on the highest (average) probability into their respective view or image-type. We used the confidence score to pick the video of the highest quality because we found that this empirically led to better performance of the algorithms. This choice was based on a precedent in literature^18^. The workflow annotates the video or image with the highest (average) confidence score of all frames. Depending on the measurement, the workflow takes the median or mean of all available beats or, in the case of TRV max, the highest value available in all images. We determined the median for an even beat number by dividing the number of measurements by two and floor the value. This means that in case of four ordered beats, we will use the second beat. In case of the mean, we summed each measured value per beat within the video and divided this by the total number of measured beats. Supplementary table 10 outlines for each measurement whether the mean, mean or highest value (for TRV max) was taken per video or per study. If multiple videos or images pass the confidence-based threshold, the workflow will select the video with the highest view confidence value. Annotation of each video frame and Doppler image is performed using CNNs based on a U-Net style architecture. Volume curves are generated based on the annotation of 2D videos. These volume curves are used to determine peak systole and diastole phases. The workflow will annotate all available beats in the 2D video or Doppler image. The measurement quality is based on a number of Boolean statements relating to the shape and placement of the annotation trace (i.e., the shape is as expected and the placement of the annotation is within expected bounds within the frame), congruency between systolic and diastolic phases (i.e., the timing of the systolic and diastolic phase identified from the volume curves are similar to those identified on ECG) or timing of the beats (i.e., if the heart rate is >120 BPM, Doppler will not be measured), and the physiological range of the quantitative results of the annotation. If any of these conditions is not met, the measurement quality is considered poor. When measurement quality is poor the workflow will not output a result^25^.

### Study design and echocardiographic studies

The deep learning workflow was installed at the Brigham & Women’s Hospital Cardiac Imaging Core Laboratory (Boston, MA). Two study cohorts with patients with HFrEF and individuals without HF were manually selected based on the in- and exclusion criteria outlined in the study protocol. There were no pre-determined criteria regarding the inclusion of participants without HF. In this study, participants without HF compromised roughly a third of the total. Patients with HFrEF were enrolled in a single-arm clinical trial (NCT02887183). Patients enrolled in this trial provided written informed consent, were men and women ≥18 years, had HFrEF (left ventricular ejection fraction <40%) and New York Heart Association (NYHA) class II-IV. All echoes were performed at baseline before starting study treatment. Additional individuals without heart failure were similarly enrolled in a separate clinical trial (NCT03767855). Participants without HF were men and women between 18 and 55 years with a body mass index (BMI) of 18–32 kg/m2, and were in good health in the opinion of the investigator and were not taking medications for the treatment of any chronic or episodic medical disease or condition. In total, 421 exams were selected from patients who had previously diagnosed HFrEF, while the additional 179 images were selected from individuals without HF.

Two expert sonographers evaluated each study once on top of previous measurement performed for other study purposes for a total of three manual evaluations per study. Annotations by the sonographers were made using using Echostation Version 5.015 (MV-Adur, RVIDd) and Version 5.014 (all other measurements). Echostation is a proprietary validated echocardiographic analysis software, which allows for all measurements to be directly input and tracked within an automated database system. The sonographers were unaware or could not see the annotation by the other sonographers or the automated workflow. Similarly, the automated workflow did not have access to any of the manual annotations. For each study, the human experts chose the best video or image and beats for their annotations according to existing ASE guidelines^11^. The deep learning workflow did not have access to the previous study annotation and the new two human annotations. The 23 echocardiographic parameters considered are listed in Table 1.

All echocardiographic images had been anonymized at the site level. Patients whose echocardiograms were utilized for this analysis provided written informed consent for providing echocardiographic images, and analysis of anonymized echocardiographic images was approved by the Mass General Brigham Institutional Review Board.

### Primary and secondary endpoints

The primary outcome was the interchangeability of deep learning and human measurements. We considered that deep learning measurements were completely interchangeable with human measurements when the variance of differences between deep learning and human measurements is no larger than the variance of differences in measurements between human experts^31^, i.e., individual bioequivalence. To assess the interchangeability of human and machine-generated measurements, we used the individual equivalence coefficient (IEC) as the study’s primary endpoint. The IEC is a scale-free measure of relative differences, helpful in assessing agreement between multiple observers^31^. The IEC can be calculated as IEC = [*Q*_*TR*_ − *Q*_*RR*_]/(*Q*_*RR*_/2). *Q*_*TR*_ is the mean of the squared differences between within-patient responses from the automated workflow and each of 3 human reference measurements, *Q*_*RR*_ is the mean of the squared differences between three pairs of within-patient reference measurements. The expected value of IEC is 0 if the differences between deep learning algorithms and human experts have the same variability as the differences between human experts. The expected value of IEC is less than 0 if the differences between deep learning algorithms and the three human experts are less variable than the differences in measurements among the three human experts.

We pre-determined the success criterion as a non-inferiority margin of **∆** = 0.25, such that automated measurements are deemed inferior to human measurements if IEC + 1.96*standard deviation (SD) of IEC > 0.25, coinciding with automated measurements having a 25% higher variance of within-patient errors than human measurements. We conducted bootstrap resampling (10,000 replicates) to estimate SD(IEC) for each echocardiographic feature.

Exploratory endpoints included measurements of agreement between automated and human expert measurements, including the interclass correlation coefficient (ICC), mean absolute deviation (MAD), within-patient coefficient of variation (wCV), concordance correlation coefficient (CCC), and limits of agreement (LOA) by Bland-Altman testing^31,32^. In secondary analysis, we calculated the ICC and standard deviation (STD) for the automated measurements of all available images and videos per study.

### Sample size calculation

To calculate the sample size for this study, we performed 10,000 simulations to estimate power at various thresholds for the SD of the IEC. We estimated that a sample size of *N* = 600 patients, would provide 80% power at a Gamma of 0.96. *N* = 600 refers to the number of participants (not images). A full explanation of the sample size calculation is provided in the supplementary material.

### Statistical analysis

Characteristics of participants are presented as the mean and SD, or number with percentages depending on the nature of the variable. The primary outcome, the individual equivalence coefficient (IEC), was calculated by comparing differences between the automated and each individual human measurement (e.g., the average of automated vs. human 1, automated vs. human 2, and automated vs human 3) against the differences for each study between the three humans (e.g, human1 vs human 2, human 1 vs human 3 and human 2 vs human 3). This means that the IEC was calculated by subtracting the mean of the squared differences between the three pairs of within-patient human measurements (Q_RR_) from the mean of the squared differences between within-patient responses from the automated workflow and each of three human measurements (Q_RR_). The ICC, MAD, wCV, CCC, LOA and CP were calculated using sklearn.metrics using mixed-effects modelling. In secondary analyses, we calculated the IEC and secondary endpoints separately for pairs of human readers and automated versus human readers and for automated versus individual human readers, where appropriate. Because the automated workflow has decision rules which exclude low-quality images or measurements, we estimated the yield per measurement of the automated workflow. The yield was the proportion of studies measured by the automated workflow among studies with three human readers, calculated as x/n, where n equals the number of participants with three reads, and x equals the number of participants with three reads and an automated read. The 95%CI of the yield was calculated using the 0.05 and 0.95 quantiles of the binomial distribution. In sensitivity analysis, we relaxed the measurement quality filters by moving all automated “low confidence” measurements into “high confidence for three of the parameters with the lowest yield. Other packages used include NumPy, pandas, seaborn, and SciPy. All analyses were performed using Python 3.8. All tests were considered two-sided.

### Reporting summary

Further information on research design is available in the Nature Research Reporting Summary linked to this article.

## Supplementary information

Supplementary Information

Reporting Summary

## Data Availability

The DICOM videos are housed at the Brigham and Women’s Hospital core laboratory. Third-party contractual agreements prohibit sharing the DICOM videos publicly. Assessment of the original videos can be made on-site at Brigham and Women’s Hospital core laboratory by request to the corresponding author and with appropriate data-use agreements.

## Code availability

The code used for calculating measures of agreement and the IEC are included in the supplementary material. The code utilized in the software is not publicly available as this is considered proprietary intellectual property by the sponsor (United States patent number 10,631.828 B1). The software will be made available by request to the sponsor (US2.AI) for the purposes of reproducing the presented results.
