# Supplementary material for: A formal validation of a deep learning-based automated workflow for the interpretation of the echocardiogram
Source: Nat Commun. 2022 Nov 9;13:6776. doi: 10.1038/s41467-022-34245-1 (PMC9646849; doi:10.1038/s41467-022-34245-1)
Supplement: Supplementary file 1 — Supplementary Information [file 41467_2022_34245_MOESM1_ESM.docx]

**Supplemental material.**

**Sample size calculation**

To calculate the sample size for this study we used the estimates of SD of the IEC from each combination of sample sizes (N=100 to 1000) and error variance ratios (Gamma = 0.5 to 1.5) to estimate power to reject the inferiority of automated measurements relative to human measurements. We simulated x=IEC as a normal random variable, with expectations equal to Gamma - 1 and SD equal to the SD(IEC). We calculated an upper confidence limit as upper confidence limit = x + SD(IEC), and we calculated an indicator reject=1 when upper confidence limit< **∆**, 0 otherwise. We ran 10,000 replicates to estimate power to reject inferiority at various thresholds. At a modest threshold of delta=0.25, we plotted power as a function of sample size and Gamma (**Supplementary Figure 3**). We selected a sample size of N=600 patients based on **Supplementary Figure 3**, providing 76% power at Gamma=1.0 (identical true error SD for automated and human measurements) and 80% power at Gamma=0.96 when the automated workflow has 4% better within-patient variance than the human measurements. N=600 refers to the number of participants (not images).

**Supplementary table 1:** Participant characteristics.

| N | 600 |
| --- | --- |
| Age mean (SD), years | 57 (16) |
| Women n (%) | 186 (69%) |
| Systolic blood pressure mean (SD), mm Hg | 120 (17) |
| Diastolic blood pressure mean (SD), mm Hg | 73 (11) |
| Heart rate mean (SD), BPM | 69 (12) |
| Heart failure patient n (%) | 421 (70%) |

Abbreviations: BPM, beats per minute; SD, standard deviation

**Supplementary table 2: Sensitivity analyses for TR Vmax, s’lateral and s’ septal relaxing measurement confidence rules.**

| Original results | | | | | | | |
| --- | --- | --- | --- | --- | --- | --- | --- |
|  | **IEC** | **IEC 95% CI** | | **n (3 reader sets)** | **n (automated + 3 reader sets)** | **yield** | **yield (95% CI)** |
|  |  | **lower** | **upper** |  |  |  |  |
| TR Vmax | -0.55 | -1.02 | -0.03 | 217 | 149 | 0.69 | 0.63-0.73 |
| s' lateral | -0.18 | -0.54 | 0.2 | 547 | 445 | 0.81 | 0.78-0.84 |
| s' septal | -0.43 | -0.79 | -0.04 | 549 | 459 | 0.84 | 0.81-0.86 |
| Reduced filter results | | | | | | | |
|  | **IEC** | **IEC 95% CI** | | **n (3 reader sets)** | **n (automated + 3 reader sets)** | **yield** | **yield (95% CI)** |
|  |  | **lower** | **upper** |  |  |  |  |
| TR Vmax | -0.43 | -0.87 | 0.00936 | 217 | 161 | 0.74 | 0.69-0.78 |
| s' lateral | 1.22 | 0.27 | 2.18 | 547 | 479 | 0.88 | 0.85-0.90 |
| s' septal | -0.18 | -0.61 | 0.24 | 549 | 499 | 0.91 | 0.89-0.93 |

**Supplementary table 3**: IEC of automated versus pairs of human measurements.

|  | **IEC** | | | | | | |
| --- | --- | --- | --- | --- | --- | --- | --- |
|  | **Automated vs.**  **All human Readers** | **Reader 1 vs. Reader 2&3** | **Reader 2 vs. Reader 1&3** | **Reader 3 vs. Reader 1&2** | **Automated vs. Human 2&3** | **Automated vs. Human 1&3** | **Automated vs. Human 1&2** |
| IVSd | -0.21 | 0.11 | -0.05 | -0.05 | -0.24 | -0.27 | -0.13 |
| LVIDd | -0.37 | 0.37 | 0.77 | -0.72 | -0.21 | -0.03 | -0.69 |
| LVIDs | -0.63 | 0.03 | 0.34 | -0.30 | -0.62 | -0.54 | -0.71 |
| LVPWd | -0.04 | 0.73 | -0.23 | -0.30 | 0.61 | -0.26 | -0.30 |
| LVEDV MOD biplane | -0.81 | 3.93 | -0.86 | -0.42 | 0.80 | -1.16 | -1.00 |
| LVESV MOD biplane | -0.79 | 4.72 | -0.84 | -0.55 | 0.40 | -1.05 | -0.87 |
| LVEF MOD biplane | -0.36 | 2.25 | -0.67 | -0.37 | 0.12 | -0.56 | -0.38 |
| LAESV MOD biplane | -0.61 | 0.60 | -0.83 | 0.83 | -0.43 | -0.87 | -0.34 |
| RA area A4C (s) | -0.18 | 0.01 | 0.18 | -0.17 | 0.01 | -0.12 | -0.40 |
| RVIDd | -0.16 | 1.73 | 0.52 | -1.02 | 1.31 | -0.36 | -0.67 |
| LVSV MOD biplane | -0.16 | 2.18 | -0.61 | -0.43 | 1.14 | -0.50 | -0.45 |
|  |  |  |  |  |  |  |  |
| MV-Adur | -0.41 | 1.32 | -1.16 | 1.46 | 0.28 | -0.93 | 0.14 |
| MV-E | -0.26 | 1.93 | 0.25 | -0.96 | 0.26 | 0.03 | -0.65 |
| MV-A | -0.17 | 1.58 | -0.40 | -0.48 | 0.30 | -0.35 | -0.25 |
| DecT | -0.15 | 0.80 | 0.89 | -0.91 | 0.57 | -0.12 | -0.55 |
| e' lateral | -0.72 | 2.00 | -0.89 | 0.06 | -0.08 | -1.03 | -0.66 |
| e' septal | -0.74 | -0.42 | -0.04 | 0.63 | -0.83 | -0.93 | -0.40 |
| E/e' mean | -0.44 | 0.49 | -0.33 | -0.05 | -0.40 | -0.63 | -0.26 |
| a' lateral | -0.58 | 0.52 | -0.60 | 0.34 | -0.38 | -0.93 | -0.30 |
| a' septal | -0.67 | 0.14 | -0.53 | 0.61 | -0.60 | -0.91 | -0.39 |
| s' lateral | -0.18 | 0.74 | -0.73 | 0.42 | 0.01 | -0.54 | 0.20 |
| s' septal | -0.43 | 0.21 | -0.71 | 0.96 | -0.40 | -0.74 | 0.08 |
| TR Vmax | -0.55 | -0.59 | 2.97 | -0.61 | -0.72 | 0.08 | -0.63 |

Abbreviations: DecT, deceleration time of early diastolic MV transmitral flow; IVSd, interventricular septal diameter end diastolic; LAESV MOD biplane, left atrial end systolic volume biplane calculation based on method of discs; LVEDV MOD biplane, left ventricular end diastolic volume biplane calculation based on method of discs; LVEF MOD biplane, left ventricular ejection fraction biplane based on method of discs; LVESV MOD biplane left ventricular end systolic volume biplane calculation based on method of discs; LVSV MOD biplane, left ventricular stroke volume biplane calculation based on method of discs; MV-A, late diastolic transmitral flow; MV-Adur, duration of late diastolic transmitral flow; MV-E, early diastolic transmitral flow; RA area a4c, right atrial area at end systole in A4C; RVIDd, right ventricular end diastolic internal diameter; TR Vmax, tricuspid regurgitation maximum velocity.

**Supplementary table 4:** Intraclass correlation coefficients (ICC) between human readers and between the automated workflow and human readers.

| **Measurement** | **Reader 1 vs Reader 2** | **Reader 1 vs Reader 3** | **Reader 2 vs Reader 3** | **Automated vs Reader 1** | **Automated vs Reader 2** | **Automated vs Reader 3** |
| --- | --- | --- | --- | --- | --- | --- |
| IVSd | 0.61 | 0.55 | 0.66 | 0.57 | 0.68 | 0.67 |
| LVIDd | 0.84 | 0.9 | 0.89 | 0.9 | 0.9 | 0.92 |
| LVIDs | 0.88 | 0.91 | 0.89 | 0.93 | 0.91 | 0.94 |
| LVPWd | 0.58 | 0.59 | 0.71 | 0.63 | 0.59 | 0.58 |
| LVEDV MOD biplane | 0.78 | 0.71 | 0.9 | 0.88 | 0.87 | 0.86 |
| LVESV MOD biplane | 0.81 | 0.77 | 0.91 | 0.86 | 0.9 | 0.91 |
| LVEF MOD biplane | 0.75 | 0.74 | 0.8 | 0.75 | 0.79 | 0.82 |
| LAESV MOD biplane | 0.87 | 0.73 | 0.84 | 0.87 | 0.91 | 0.84 |
| RA area A4C (s) | 0.89 | 0.89 | 0.89 | 0.92 | 0.9 | 0.88 |
| RVIDd | 0.41 | 0.62 | 0.66 | 0.72 | 0.41 | 0.63 |
| LVSV MOD biplane | 0.28 | 0.2 | 0.65 | 0.44 | 0.58 | 0.53 |
| MV-Adur | 0.42 | -0.02 | 0.54 | 0.24 | 0.48 | 0.34 |
| MV-E | 0.94 | 0.96 | 0.98 | 0.95 | 0.97 | 0.97 |
| MV-A | 0.96 | 0.96 | 0.98 | 0.96 | 0.97 | 0.98 |
| DecT | 0.34 | 0.49 | 0.63 | 0.39 | 0.35 | 0.53 |
| e' lateral | 0.92 | 0.88 | 0.95 | 0.94 | 0.96 | 0.95 |
| e' septal | 0.92 | 0.9 | 0.89 | 0.95 | 0.92 | 0.95 |
| E/e' mean | 0.94 | 0.92 | 0.94 | 0.94 | 0.95 | 0.96 |
| a' lateral | 0.88 | 0.84 | 0.9 | 0.9 | 0.9 | 0.93 |
| a' septal | 0.9 | 0.86 | 0.9 | 0.92 | 0.92 | 0.93 |
| s' lateral | 0.96 | 0.94 | 0.96 | 0.95 | 0.96 | 0.96 |
| s' septal | 0.94 | 0.91 | 0.93 | 0.94 | 0.95 | 0.94 |
| TR Vmax | 0.87 | 0.94 | 0.88 | 0.94 | 0.89 | 0.95 |

Abbreviations: DecT, deceleration time of early diastolic MV transmitral flow; IVSd, interventricular septal diameter end diastolic; LAESV MOD biplane, left atrial end systolic volume biplane calculation based on method of discs; LVEDV MOD biplane, left ventricular end diastolic volume biplane calculation based on method of discs; LVEF MOD biplane, left ventricular ejection fraction biplane based on method of discs; LVESV MOD biplane left ventricular end systolic volume biplane calculation based on method of discs; LVIDd, left ventricular internal diameter at end diastole; LVIDs left ventricular internal diameter at end systole. LVPWd, left ventricular posterior wall thickness measured end diastolic; LVSV MOD biplane, left ventricular stroke volume biplane calculation based on method of discs; MV-A, late diastolic transmitral flow; MV-Adur, duration of late diastolic transmitral flow; MV-E, early diastolic transmitral flow; RA area a4c, right atrial area at end systole in A4C; RVIDd, right ventricular end diastolic internal diameter; TR Vmax, tricuspid regurgitation maximum velocity.

**Supplementary table 5:** Mean absolute deviation between human readers and between the automated workflow and human readers.

| **Measurement** | **Reader 1 vs Reader 2** | **Reader 1 vs Reader 3** | **Reader 2 vs Reader 3** | **Automated vs Reader 1** | **Automated vs Reader 2** | **Automated vs Reader 3** |
| --- | --- | --- | --- | --- | --- | --- |
| IVSd | 1.24 | 1.24 | 1.13 | 1.26 | 1.14 | 1.05 |
| LVIDd | 3.43 | 2.71 | 2.76 | 2.88 | 2.83 | 2.59 |
| LVIDs | 3.82 | 3.47 | 3.52 | 3.01 | 3.17 | 2.65 |
| LVPWd | 1.24 | 1.19 | 1.05 | 1.07 | 1.25 | 1.17 |
| LVEDV MOD biplane | 30.76 | 35.11 | 16.92 | 21.12 | 20.57 | 21.69 |
| LVESV MOD biplane | 22.94 | 24.67 | 11.83 | 19.42 | 13.97 | 13.26 |
| LVEF MOD biplane | 8.51 | 8.87 | 5.47 | 8.72 | 5.8 | 5.68 |
| LAESV MOD biplane | 9.9 | 13.79 | 10.64 | 9.68 | 7.97 | 10.08 |
| RA area A4C (s) | 1.96 | 1.78 | 1.85 | 1.64 | 1.85 | 1.98 |
| RVIDd | 6.77 | 4.93 | 4.2 | 4.07 | 6.24 | 4.38 |
| LVSV MOD biplane | 14.77 | 15.81 | 9.06 | 12.84 | 11.88 | 12.71 |
| MV-Adur | 26.83 | 46.08 | 25.83 | 32 | 26.78 | 31.18 |
| MV-E | 5.8 | 4.85 | 3.21 | 6.04 | 3.8 | 3.55 |
| MV-A | 5.02 | 4.79 | 3.02 | 5.3 | 3.45 | 3.18 |
| DecT | 45.11 | 33.12 | 30.65 | 32.93 | 39.22 | 29.12 |
| e' lateral | 0.95 | 1.28 | 0.78 | 0.93 | 0.72 | 0.76 |
| e' septal | 0.59 | 0.79 | 0.74 | 0.53 | 0.58 | 0.6 |
| E/e' mean | 1.17 | 1.28 | 1.38 | 1.3 | 1.33 | 1.13 |
| a' lateral | 0.73 | 1.01 | 0.72 | 0.75 | 0.59 | 0.62 |
| a' septal | 0.56 | 0.84 | 0.68 | 0.58 | 0.52 | 0.55 |
| s' lateral | 0.52 | 0.68 | 0.43 | 0.62 | 0.47 | 0.46 |
| s' septal | 0.36 | 0.55 | 0.37 | 0.43 | 0.34 | 0.37 |
| TR Vmax | 0.16 | 0.13 | 0.16 | 0.12 | 0.15 | 0.11 |

Abbreviations: DecT, deceleration time of early diastolic MV transmitral flow; IVSd, interventricular septal diameter end diastolic; LAESV MOD biplane, left atrial end systolic volume biplane calculation based on method of discs; LVEDV MOD biplane, left ventricular end diastolic volume biplane calculation based on method of discs; LVEF MOD biplane, left ventricular ejection fraction biplane based on method of discs; LVESV MOD biplane left ventricular end systolic volume biplane calculation based on method of discs; LVIDd, left ventricular internal diameter at end diastole; LVIDs left ventricular internal diameter at end systole. LVPWd, left ventricular posterior wall thickness measured end diastolic; LVSV MOD biplane, left ventricular stroke volume biplane calculation based on method of discs; MV-A, late diastolic transmitral flow; MV-Adur, duration of late diastolic transmitral flow; MV-E, early diastolic transmitral flow; RA area a4c, right atrial area at end systole in A4C; RVIDd, right ventricular end diastolic internal diameter; TR Vmax, tricuspid regurgitation maximum velocity.

**Supplementary table 6:** Within coefficient of variation (wCV) between human readers and between the automated workflow and human readers.

| **Measurement** | **Reader 1 vs Reader 2** | **Reader 1 vs Reader 3** | **Reader 2 vs Reader 3** | **Automated vs Reader 1** | **Automated vs Reader 2** | **Automated vs Reader 3** |
| --- | --- | --- | --- | --- | --- | --- |
| IVSd | 11.4 | 11.69 | 11.54 | 11.47 | 11.2 | 10.58 |
| LVIDd | 6.52 | 5.02 | 5.39 | 5.23 | 5.39 | 4.84 |
| LVIDs | 8.5 | 7.76 | 8.05 | 6.73 | 6.93 | 6.43 |
| LVPWd | 11.69 | 11.61 | 10.25 | 9.99 | 11.47 | 11.5 |
| LVEDV MOD biplane | 20.83 | 23.38 | 14.61 | 13.79 | 15.82 | 16.41 |
| LVESV MOD biplane | 27.48 | 29.64 | 17.82 | 22.3 | 18.93 | 17.94 |
| LVEF MOD biplane | 16.68 | 17.99 | 12.08 | 17.24 | 12.08 | 12.29 |
| LAESV MOD biplane | 15.35 | 22.04 | 17.64 | 15.46 | 12.99 | 17.31 |
| RA area A4C (s) | 12.67 | 12.4 | 12.4 | 10.4 | 11.63 | 12.53 |
| RVIDd | 17.3 | 12.44 | 11.76 | 10.56 | 16.64 | 11.74 |
| LVSV MOD biplane | 25.31 | 27.27 | 20.5 | 21.23 | 22.51 | 24.25 |
| MV-Adur | 17.42 | 25.4 | 15.29 | 19.03 | 15.64 | 17.83 |
| MV-E | 8.29 | 6.63 | 5.21 | 7.64 | 5.73 | 5.31 |
| MV-A | 7.53 | 7.47 | 5.46 | 7.66 | 6.14 | 5.52 |
| DecT | 20.5 | 15.9 | 14.5 | 16.17 | 19.29 | 14.74 |
| e' lateral | 11.95 | 13.95 | 8.75 | 10.59 | 8.57 | 8.8 |
| e' septal | 11.46 | 12.27 | 12.88 | 9.05 | 11.01 | 8.9 |
| E/e' mean | 14.94 | 16.41 | 14.33 | 14.95 | 13.61 | 12.03 |
| a' lateral | 10.1 | 11.57 | 9.31 | 9.11 | 9.14 | 7.74 |
| a' septal | 8.87 | 10.37 | 9.02 | 7.84 | 7.78 | 7.42 |
| s' lateral | 7.11 | 8.56 | 6.54 | 7.93 | 6.62 | 6.47 |
| s' septal | 6.63 | 8.54 | 7.05 | 7.05 | 6.24 | 6.42 |
| TR Vmax | 7.3 | 4.66 | 7.22 | 4.97 | 6.81 | 4.41 |

Abbreviations: DecT, deceleration time of early diastolic MV transmitral flow; IVSd, interventricular septal diameter end diastolic; LAESV MOD biplane, left atrial end systolic volume biplane calculation based on method of discs; LVEDV MOD biplane, left ventricular end diastolic volume biplane calculation based on method of discs; LVEF MOD biplane, left ventricular ejection fraction biplane based on method of discs; LVESV MOD biplane left ventricular end systolic volume biplane calculation based on method of discs; LVIDd, left ventricular internal diameter at end diastole; LVIDs left ventricular internal diameter at end systole. LVPWd, left ventricular posterior wall thickness measured end diastolic; LVSV MOD biplane, left ventricular stroke volume biplane calculation based on method of discs; MV-A, late diastolic transmitral flow; MV-Adur, duration of late diastolic transmitral flow; MV-E, early diastolic transmitral flow; RA area a4c, right atrial area at end systole in A4C; RVIDd, right ventricular end diastolic internal diameter; TR Vmax, tricuspid regurgitation maximum velocity.

**Supplementary table 7:** Root mean squared error (RMSE) between human readers and between the automated workflow and human readers.

| **Measurement** | **Reader 1 vs Reader 2** | **Reader 1 vs Reader 3** | **Reader 2 vs Reader 3** | **Automated vs Reader 1** | **Automated vs Reader 2** | **Automated vs Reader 3** |
| --- | --- | --- | --- | --- | --- | --- |
| IVSd | 1.16 | 1.16 | 1.13 | 1.14 | 1.1 | 1.01 |
| LVIDd | 3.4 | 2.65 | 2.8 | 2.74 | 2.77 | 2.52 |
| LVIDs | 3.59 | 3.23 | 3.39 | 2.83 | 2.95 | 2.69 |
| LVPWd | 1.15 | 1.14 | 0.98 | 1 | 1.12 | 1.12 |
| LVEDV MOD biplane | 27.07 | 29.71 | 16.51 | 18.86 | 19.42 | 19.67 |
| LVESV MOD biplane | 21.97 | 23.41 | 12.39 | 18.62 | 14.01 | 13.11 |
| LVEF MOD biplane | 7.15 | 7.59 | 5.05 | 7.48 | 5.2 | 5.21 |
| LAESV MOD biplane | 9.51 | 12.62 | 9.81 | 9.48 | 7.75 | 9.51 |
| RA area A4C (s) | 1.85 | 1.75 | 1.8 | 1.54 | 1.77 | 1.84 |
| RVIDd | 5.69 | 4.26 | 3.68 | 3.74 | 5.39 | 3.97 |
| LVSV MOD biplane | 12.68 | 13.14 | 8.94 | 11.36 | 11.01 | 11.39 |
| MV-Adur | 24.71 | 38.54 | 25.12 | 26.95 | 24.11 | 29.26 |
| MV-E | 6.33 | 5.02 | 4.08 | 5.82 | 4.51 | 4.14 |
| MV-A | 5.02 | 4.95 | 3.73 | 5.12 | 4.22 | 3.78 |
| DecT | 40.83 | 29.91 | 30.25 | 29.6 | 39.27 | 28.37 |
| e' lateral | 1.04 | 1.25 | 0.81 | 0.92 | 0.78 | 0.82 |
| e' septal | 0.74 | 0.82 | 0.88 | 0.59 | 0.73 | 0.61 |
| E/e' mean | 1.79 | 1.88 | 1.65 | 1.75 | 1.59 | 1.34 |
| a' lateral | 0.8 | 0.94 | 0.78 | 0.72 | 0.74 | 0.65 |
| a' septal | 0.63 | 0.76 | 0.68 | 0.56 | 0.57 | 0.56 |
| s' lateral | 0.51 | 0.63 | 0.49 | 0.58 | 0.49 | 0.49 |
| s' septal | 0.38 | 0.5 | 0.42 | 0.4 | 0.37 | 0.38 |
| TR Vmax | 0.19 | 0.12 | 0.19 | 0.13 | 0.18 | 0.11 |

Abbreviations: DecT, deceleration time of early diastolic MV transmitral flow; IVSd, interventricular septal diameter end diastolic; LAESV MOD biplane, left atrial end systolic volume biplane calculation based on method of discs; LVEDV MOD biplane, left ventricular end diastolic volume biplane calculation based on method of discs; LVEF MOD biplane, left ventricular ejection fraction biplane based on method of discs; LVESV MOD biplane left ventricular end systolic volume biplane calculation based on method of discs; LVIDd, left ventricular internal diameter at end diastole; LVIDs left ventricular internal diameter at end systole. LVPWd, left ventricular posterior wall thickness measured end diastolic; LVSV MOD biplane, left ventricular stroke volume biplane calculation based on method of discs; MV-A, late diastolic transmitral flow; MV-Adur, duration of late diastolic transmitral flow; MV-E, early diastolic transmitral flow; RA area a4c, right atrial area at end systole in A4C; RVIDd, right ventricular end diastolic internal diameter; TR Vmax, tricuspid regurgitation maximum velocity.

**Supplementary table 8:** Correlation coefficient between human readers and between the automated workflow and human readers.

| **Measurement** | **Reader 1 vs Reader 2** | **Reader 1 vs Reader 3** | **Reader 2 vs Reader 3** | **Automated vs Reader 1** | **Automated vs Reader 2** | **Automated vs Reader 3** |
| --- | --- | --- | --- | --- | --- | --- |
| IVSd | 0.64 | 0.62 | 0.69 | 0.63 | 0.7 | 0.67 |
| LVIDd | 0.86 | 0.91 | 0.9 | 0.91 | 0.9 | 0.92 |
| LVIDs | 0.89 | 0.91 | 0.9 | 0.93 | 0.92 | 0.94 |
| LVPWd | 0.61 | 0.63 | 0.72 | 0.64 | 0.61 | 0.61 |
| LVEDV MOD biplane | 0.89 | 0.9 | 0.91 | 0.92 | 0.9 | 0.93 |
| LVESV MOD biplane | 0.9 | 0.9 | 0.92 | 0.91 | 0.91 | 0.93 |
| LVEF MOD biplane | 0.84 | 0.8 | 0.81 | 0.79 | 0.81 | 0.83 |
| LAESV MOD biplane | 0.88 | 0.88 | 0.93 | 0.88 | 0.92 | 0.92 |
| RA area A4C (s) | 0.9 | 0.9 | 0.91 | 0.94 | 0.9 | 0.91 |
| RVIDd | 0.7 | 0.72 | 0.75 | 0.73 | 0.62 | 0.69 |
| LVSV MOD biplane | 0.43 | 0.5 | 0.68 | 0.47 | 0.65 | 0.72 |
|  |  |  |  |  |  |  |
| MV-Adur | 0.71 | 0.55 | 0.66 | 0.5 | 0.48 | 0.45 |
| MV-E | 0.96 | 0.97 | 0.98 | 0.96 | 0.97 | 0.98 |
| MV-A | 0.97 | 0.97 | 0.98 | 0.97 | 0.97 | 0.98 |
| DecT | 0.65 | 0.58 | 0.73 | 0.41 | 0.55 | 0.56 |
| e' lateral | 0.93 | 0.93 | 0.96 | 0.95 | 0.96 | 0.96 |
| e' septal | 0.93 | 0.94 | 0.91 | 0.96 | 0.93 | 0.96 |
| E/e' mean | 0.94 | 0.94 | 0.96 | 0.94 | 0.95 | 0.96 |
| a' lateral | 0.9 | 0.92 | 0.92 | 0.93 | 0.91 | 0.94 |
| a' septal | 0.91 | 0.93 | 0.92 | 0.94 | 0.93 | 0.94 |
| s' lateral | 0.96 | 0.96 | 0.97 | 0.96 | 0.96 | 0.96 |
| s' septal | 0.96 | 0.96 | 0.95 | 0.96 | 0.95 | 0.95 |
| TR Vmax | 0.89 | 0.95 | 0.88 | 0.95 | 0.89 | 0.96 |

Abbreviations: DecT, deceleration time of early diastolic MV transmitral flow; IVSd, interventricular septal diameter end diastolic; LAESV MOD biplane, left atrial end systolic volume biplane calculation based on method of discs; LVEDV MOD biplane, left ventricular end diastolic volume biplane calculation based on method of discs; LVEF MOD biplane, left ventricular ejection fraction biplane based on method of discs; LVESV MOD biplane left ventricular end systolic volume biplane calculation based on method of discs; LVIDd, left ventricular internal diameter at end diastole; LVIDs left ventricular internal diameter at end systole. LVPWd, left ventricular posterior wall thickness measured end diastolic; LVSV MOD biplane, left ventricular stroke volume biplane calculation based on method of discs; MV-A, late diastolic transmitral flow; MV-Adur, duration of late diastolic transmitral flow; MV-E, early diastolic transmitral flow; RA area a4c, right atrial area at end systole in A4C; RVIDd, right ventricular end diastolic internal diameter; TR Vmax, tricuspid regurgitation maximum velocity.

**Supplementary table 9:** Interclass correlation coefficient and standard deviation for all monoplane measurements for all repeated videos and images passing the confidence threshold within each available study.

|  | **ICC** | **STD** |
| --- | --- | --- |
| **Measurement** |  |  |
| **IVSd (mm)** | 0.96 | 1.67 |
| **LVIDd (mm)** | 0.95 | 1.6 |
| **LVIDs (mm)** | 0.79 | 0.6 |
| **LVEDV Monoplane A4C (ml)** | 0.79 | 15.68 |
| **LVESV Monoplane A4C (mL)** | 0.81 | 12.65 |
| **LVEF Monoplane A4C (%)** | 0.74 | 5.23 |
| **LAESV Monoplane A4C (ml)** | 0.88 | 5.79 |
| **RA area A4C (s) (cm^2^)** | 0.89 | 1.35 |
| **RVIDd (mm)** | 0.75 | 2.88 |
| **LVSV MOD biplane (ml)** | 0.54 | 9.3 |
| **MV-Adur (ms)** | 0.73 | 16.32 |
| **MV-E (cm/s)** | 0.94 | 5.05 |
| **MV-A (cm/s)** | 0.94 | 5.09 |
| **DecT (ms)** | 0.52 | 24.22 |
| **e' lateral (cm/s)** | 0.96 | 0.56 |
| **e' septal (cm/s)** | 0.9 | 0.6 |
| **a' lateral (cm/s)** | 0.94 | 0.5 |
| **a' septal (cm/s)** | 0.92 | 0.49 |
| **s' lateral (cm/s)** | 0.95 | 0.45 |
| **s' septal (cm/s)** | 0.91 | 0.37 |
| **TR Vmax (m/s)** | 0.92 | 0.12 |

Abbreviations: DecT, deceleration time of early diastolic MV transmitral flow; IVSd, interventricular septal diameter end diastolic; LAESV MOD biplane, left atrial end systolic volume biplane calculation based on method of discs; LVEDV MOD biplane, left ventricular end diastolic volume biplane calculation based on method of discs; LVEF MOD biplane, left ventricular ejection fraction biplane based on method of discs; LVESV MOD biplane left ventricular end systolic volume biplane calculation based on method of discs; LVIDd, left ventricular internal diameter at end diastole; LVIDs left ventricular internal diameter at end systole. LVPWd, left ventricular posterior wall thickness measured end diastolic; LVSV MOD biplane, left ventricular stroke volume biplane calculation based on method of discs; MV-A, late diastolic transmitral flow; MV-Adur, duration of late diastolic transmitral flow; MV-E, early diastolic transmitral flow; RA area a4c, right atrial area at end systole in A4C; RVIDd, right ventricular end diastolic internal diameter; STD, standard deviation; TR Vmax, tricuspid regurgitation maximum velocity.

**Supplementary Table 10:** Table outlining the method for computing each measurement. Methods include the mean or median for each highest quality video, a computed (calculated) measurement, or the maximum value of all available images.

| **Parameter** | **Method** |
| --- | --- |
| RA area A4C (systole) | Mean |
| RVIDd | Median |
| LVEF MOD monoplane (volume) | Computed |
| LVEDV MOD monoplane (volume) | Median |
| LVESV MOD monoplane (volume) | Median |
| LVSV MOD monoplane (volume) | Computed |
| LAESV MOD monoplane (volume) | Median |
| LVEF MOD monoplane (volume) | Computed |
| LVEDV MOD monoplane (volume) | Median |
| LVESV MOD (monoplane volume) | Median |
| LVSV MOD (monoplane volume) | Computed |
| LAESV MOD (monoplane volume) | Median |
| LVID (systole and diastole) | Mean |
| IVSD (diastole) | Mean |
| LVPW (diastole) | Mean |
| TR Vmax | Maximum value |
| MV-Adur | Mean |
| MV-E | Mean |
| MV-A | Mean |
| DecT | Mean |
| E' lateral | Mean |
| E' septal | Mean |
| A' lateral | Mean |
| A' septal | Mean |
| S' lateral | Mean |
| S' septal | Mean |
| E / e' mean | Computed |

**Supplementary figure 1:** Bland-Altman plots for LVEF (A), LAESV (B), e’ lateral (C) and E/e’ mean (D)


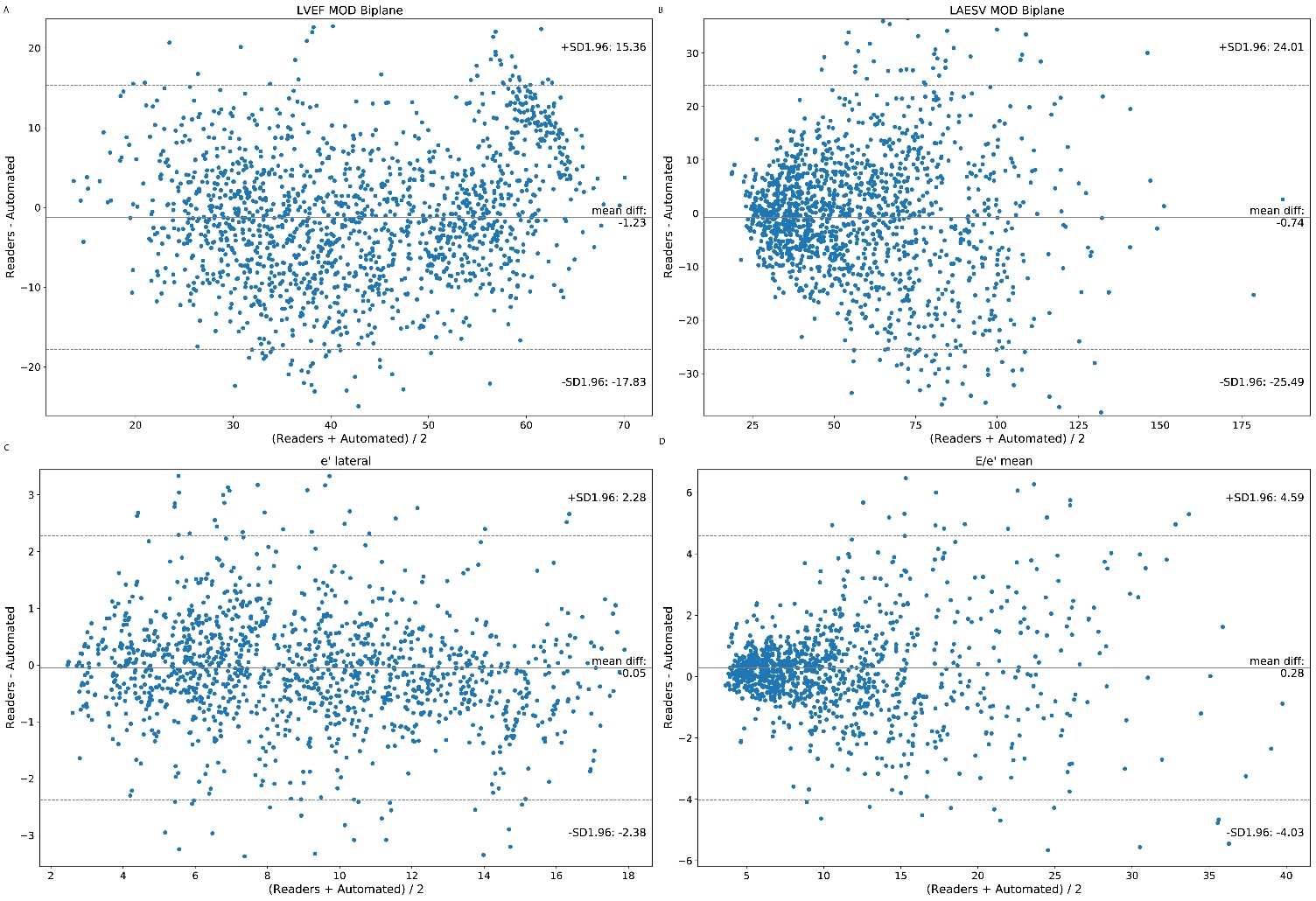


**Supplementary figure2:** Overview of the automated workflow

**
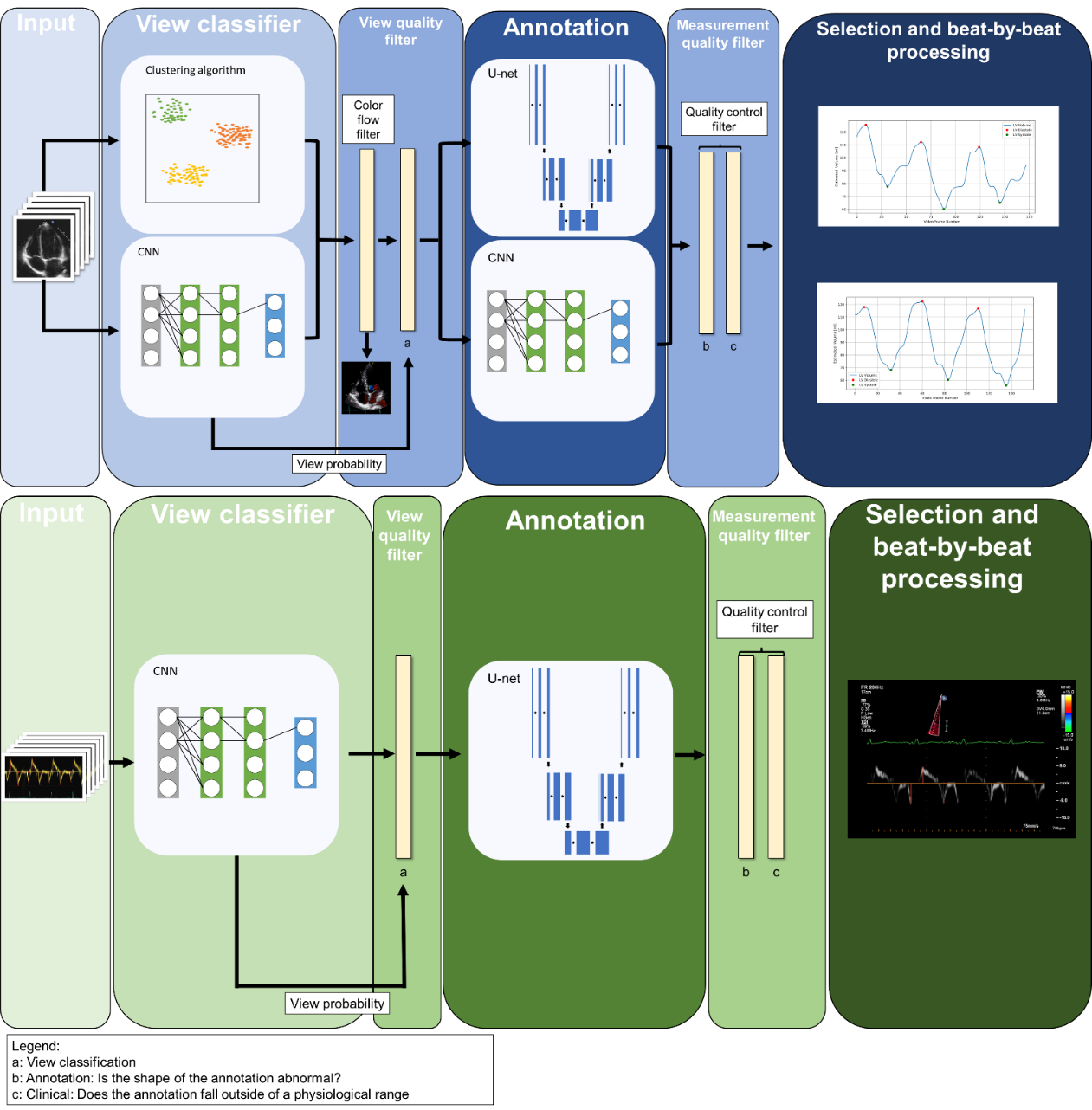
**

**Supplementary figure 3:** Sample sizes for different according to gamma and power.


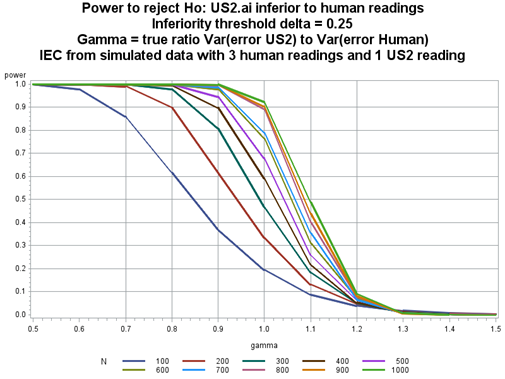


**References**
