# Supplementary material for: A formal validation of a deep learning-based automated workflow for the interpretation of the echocardiogram
Source: Nat Commun. 2022 Nov 9;13:6776. doi: 10.1038/s41467-022-34245-1 (PMC9646849; doi:10.1038/s41467-022-34245-1)
Supplement: Supplementary file 2 — Reporting Summary [file 41467_2022_34245_MOESM2_ESM.pdf]

nature portfolio

Corresponding author(s): Scott D Solomon

Last updated by author(s): Oct 3, 2022

Reporting Summary

Nature Portfolio wishes to improve the reproducibility of the work that we publish. This form provides structure for consistency and transparency in reporting. For further information on Nature Portfolio policies, see our Editorial Policies and the Editorial Policy Checklist.

Statistics

For all statistical analyses, confirm that the following items are present in the figure legend, table legend, main text, or Methods section.

n/a	Confirmed	
☐	☒	The exact sample size (*n*) for each experimental group/condition, given as a discrete number and unit of measurement
☐	☒	A statement on whether measurements were taken from distinct samples or whether the same sample was measured repeatedly
☐	☒	The statistical test(s) used AND whether they are one- or two-sided<br>*Only common tests should be described solely by name; describe more complex techniques in the Methods section.*
☐	☒	A description of all covariates tested
☐	☒	A description of any assumptions or corrections, such as tests of normality and adjustment for multiple comparisons
☐	☒	A full description of the statistical parameters including central tendency (e.g. means) or other basic estimates (e.g. regression coefficient) AND variation (e.g. standard deviation) or associated estimates of uncertainty (e.g. confidence intervals)
☐	☒	For null hypothesis testing, the test statistic (e.g. *F*, *t*, *r*) with confidence intervals, effect sizes, degrees of freedom and *P* value noted<br>*Give P values as exact values whenever suitable.*
☒	☐	For Bayesian analysis, information on the choice of priors and Markov chain Monte Carlo settings
☒	☐	For hierarchical and complex designs, identification of the appropriate level for tests and full reporting of outcomes
☐	☒	Estimates of effect sizes (e.g. Cohen's *d*, Pearson's *r*), indicating how they were calculated

Our web collection on statistics for biologists contains articles on many of the points above.

Software and code

Policy information about availability of computer code

Data collection	The code used for calculating measures of agreement and the IEC are included in the supplementary material. The code utilized in the software is not publicly available as this is considered proprietary intellectual property by the sponsor (United States patent number 10,631.828 B1). The software will be made available by request to the sponsor (US2.AI) for the purposes of reproducing the presented results. Annotations by the sonographers were made using using Echostation Version 5.015 (MV-Adur, RVIDd) and Version 5.014 (all other measurements). Echostation is a proprietary validated echocardiographic analysis software, which allows for all measurements to be directly input and tracked within an automated database system.
Data analysis	No software was used

For manuscripts utilizing custom algorithms or software that are central to the research but not yet described in published literature, software must be made available to editors and reviewers. We strongly encourage code deposition in a community repository (e.g. GitHub). See the Nature Portfolio guidelines for submitting code & software for further information.

Data

Policy information about availability of data

All manuscripts must include a data availability statement. This statement should provide the following information, where applicable:

- Accession codes, unique identifiers, or web links for publicly available datasets
- A description of any restrictions on data availability
- For clinical datasets or third party data, please ensure that the statement adheres to our policy

The DICOM videos are housed at the Brigham and Women's Hospital core laboratory. Third-party contractual agreements prohibit sharing the DICOM videos publicly. Assessment of the original videos can be made on-site at Brigham and Women's Hospital core laboratory by request to the corresponding author and with appropriate data-use agreements.

Human research participants

Policy information about studies involving human research participants and Sex and Gender in Research.

Reporting on sex and gender	186 (69%) were women, findings apply to both men and women. Sex/gender was considered based on physician and self reporting. We did not report sex stratified analyses since there was no indication of effect modification by sex.
Population characteristics	The mean age 602 participants were 57 (± 16) years, 186 (69%) were women, and 421 (70%) had HFrEF. The mean systolic blood pressure was 120 (± 17) mm Hg. The mean LVEF was 42% (±14%), the mean E/e' was 12 (± 7).
Recruitment	Participants were recruited as part of randomized controlled clinical trials (NCT02887183 and NCT03767855), which might have caused selection bias.
Ethics oversight	Mass General Brigham Institutional Review Board approved the study

Note that full information on the approval of the study protocol must also be provided in the manuscript.

Field-specific reporting

Please select the one below that is the best fit for your research. If you are not sure, read the appropriate sections before making your selection.

[x] Life sciences [ ] Behavioural & social sciences [ ] Ecological, evolutionary & environmental sciences

For a reference copy of the document with all sections, see nature.com/documents/nr-reporting-summary-flat.pdf

Life sciences study design

All studies must disclose on these points even when the disclosure is negative.

Sample size	To calculate the sample size for this study, we performed 10,000 simulations to estimate power at various thresholds for the SD of the IEC. We estimated that a sample size of N=600 patients, would provide 80% power at a Gamma of 0.96. N=600 refers to the number of participants (not images). A full explanation of the sample size calculation is provided in the supplementary material.
Data exclusions	Patients with HFrEF were enrolled in a single-arm clinical trial (NCT02887183). Patients enrolled in this trial provided written informed consent, were men and women ≥18 years, had HFrEF (left ventricular ejection fraction <40%) and New York Heart Association (NYHA) class II-IV. All echoes were performed at baseline before starting study treatment. Additional individuals without heart failure were similarly enrolled in a separate clinical trial (NCT03767855). Participants without heart failure were men and women between 18 and 55 years with a body mass index (BMI) of 18-32 kg/m2, and were in good health in the opinion of the investigator and were not taking medications for the treatment of any chronic or episodic medical disease or condition. In total, 421 exams were selected from patients who had previously diagnosed HFrEF, while the additional 179 images were selected from individuals without HF.
Replication	Automated measurements were compared against individual measurements of three expert sonographer for each study.
Randomization	Randomization was not applicable to our study because it was not a randomized controlled trial.
Blinding	The human readers and automated workflow were unaware of each other's measurements and annotations.

Reporting for specific materials, systems and methods

We require information from authors about some types of materials, experimental systems and methods used in many studies. Here, indicate whether each material, system or method listed is relevant to your study. If you are not sure if a list item applies to your research, read the appropriate section before selecting a response.

Materials & experimental systems

n/a	Involved in the study
☒	☐ Antibodies
☒	☐ Eukaryotic cell lines
☒	☐ Palaeontology and archaeology
☒	☐ Animals and other organisms
☐	☒ Clinical data
☒	☐ Dual use research of concern

Methods

n/a	Involved in the study
☒	☐ ChIP-seq
☒	☐ Flow cytometry
☒	☐ MRI-based neuroimaging

Clinical data

Policy information about clinical studies

All manuscripts should comply with the ICMJEguidelines for publication of clinical research and a completedCONSORT checklist must be included with all submissions.

Clinical trial registration: NA

Study protocol: The study protocol was made available to reviewers

Data collection: NA

Outcomes: The primary outcome was the interchangeability of deep learning and human measurements. We considered that deep learning measurements were completely interchangeable with human measurements when the variance of differences between deep learning and human measurements is no larger than the variance of differences in measurements between human experts. To assess the interchangeability of human and machine-generated measurements, we used the individual equivalence coefficient (IEC) as the study's primary endpoint. The IEC is a scale-free measure of relative differences, helpful in assessing agreement between multiple observers. The IEC can be calculated as IEC = [QTR - QRR]/(QRR/2). QTR is the mean of the squared differences between within-patient responses from Us2.v1 and each of 3 human reference measurements, QRR is the mean of the squared differences between 3 pairs of within-patient reference measurements. The expected value of IEC is 0 if the differences between deep learning algorithms and human experts have the same variability as the differences between human experts. The expected value of IEC is less than 0 if the differences between deep learning algorithms and the three human experts are less variable than the differences in measurements among the three human experts.
